# Natural antisense transcripts with coding capacity in *Arabidopsis *may have a regulatory role that is not linked to double-stranded RNA degradation

**DOI:** 10.1186/gb-2005-6-6-r51

**Published:** 2005-06-01

**Authors:** Chih-Hung Jen, Ioannis Michalopoulos, David R Westhead, Peter Meyer

**Affiliations:** 1 School of Biochemistry and Microbiology, University of Leeds, Leeds LS2 9JT, UK; 2 Centre for Plant Science, The University of Leeds, Leeds LS2 9JT, UK

## Abstract

**Background:**

Overlapping transcripts in antisense orientation have the potential to form double-stranded RNA (dsRNA), a substrate for a number of different RNA-modification pathways. One prominent route for dsRNA is its breakdown by Dicer enzyme complexes into small RNAs, a pathway that is widely exploited by RNA interference technology to inactivate defined genes in transgenic lines. The significance of this pathway for endogenous gene regulation remains unclear.

**Results:**

We have examined transcription data for overlapping gene pairs in *Arabidopsis thaliana*. On the basis of an analysis of transcripts with coding regions, we find the majority of overlapping gene pairs to be convergently overlapping pairs (COPs), with the potential for dsRNA formation. In all tissues, COP transcripts are present at a higher frequency compared to the overall gene pool. The probability that both the sense and antisense copy of a COP are co-transcribed matches the theoretical value for coexpression under the assumption that the expression of one partner does not affect the expression of the other. Among COPs, we observe an over-representation of spliced (intron-containing) genes (90%) and of genes with alternatively spliced transcripts. For loci where antisense transcripts overlap with sense transcript introns, we also find a significant bias in favor of alternative splicing and variation of polyadenylation.

**Conclusion:**

The results argue against a predominant RNA degradation effect induced by dsRNA formation. Instead, our data support alternative roles for dsRNAs. They suggest that at least for a subgroup of COPs, antisense expression may induce alternative splicing or polyadenylation.

## Abstract 2

Transcription data analysis of overlapping gene pairs in *Arabidopsis thaliana *argues against a predominant RNA degradation effect induced by dsRNA formation. Instead, it suggests alternative roles for dsRNAs such as regulation of alternative splicing in polyadenylation.

## Background

Genome-wide searches in the genomes of several species have identified a surprisingly high proportion of overlapping gene pairs. Depending on the sample sizes analyzed and search criteria, the frequencies for overlapping gene pairs vary between 4% and 9% for the human genome, 1.7%-14% in the murine genome, and up to 22% in the fly genome [1]. The predominant composition of overlapping gene pairs is an antiparallel convergent arrangement [2,3], where sense and antisense genes overlap within their 3' regions. Joint expression of both these genes in the same cell would allow the partly overlapping transcripts to associate as dsRNA molecules, which may interfere with RNA processing, transport, stability or other molecular mechanisms. Convergently overlapping gene pairs (COPs) can therefore provide the source for natural antisense transcripts (NATs) that may act as regulators of the sense gene. In addition to NATs being transcribed from the same locus as the sense transcript (*cis*-NATs), NATs can be transcribed from a different locus (*trans*-NATs), as illustrated by a search for overlapping transcripts with coding capacity in the human genome, which identified 87 *cis*-NATs and 80 *trans-*NATs [3].

In bacteria, more than 100 NATs are involved in the regulation of a variety of biological functions, including the control of copy number, conjugation and post-segregational killing in plasmids, lysis/lysogeny switches in phages, and transposition frequency in transposons [4]. In eukaryotes, a very detailed characterization of the molecular role of specific NATs has only been achieved for a few examples.

NAT-mediated interference with splicing is illustrated by the alternative processing of mRNAs of the gene for the thyroid hormone receptor ErbAα, which is regulated by an antisense transcript [5]. Overlapping genes can share a bidirectional poly(A) region as demonstrated for the human genes *ABHD1 *and *Sec12 *[6]. Several examples document the fact that antisense transcripts can increase sense transcript stability, when dsRNA regions cover the 3' untranslated region (UTR) and possibly mask out target sequences for RNA cleavage [7]. Alternatively, RNA duplex formation can increase transcript sensitivity and induce site-specific cleavage, as shown for the human *TYMS *mRNA and *TRS *antisense transcripts [8].

An example of RNA interference (RNAi)-based regulation of an endogenous gene via NATs is the repression of the testis-expressed *Stellate *gene in *Drosophila *by paralogous *Su(Ste) *tandem repeats [9]. Both strands of repressor *Su(Ste) *repeats are transcribed, producing sense and antisense RNA, most probably as part of a dsRNA-based silencing mechanism, as *Stellate *silencing is associated with the presence of short *Su(Ste) *RNAs. Antisense expression can also affect translation, as illustrated by the influence of an antisense transcript on the translation of different isoforms of fibroblast growth factor-2 (FGF2) [10]. In the nucleus, dsRNA can be edited by dsRNA-dependent adenosine deaminases, which convert about 50% of adenosine residues into inosines, leading to the unwinding of the RNA duplex [11]. Inosine-containing RNAs are not translated as they are retained in the nucleus [12].

In mice about 35% of overlapping genes transcribe noncoding RNA. Overlapping genes are scattered around the genome with no apparent bias. Overlaps range from 20 to 3,400 base-pairs (bp) with an average of 372 bp, as far as the quality of transcript annotation allows such predictions. There is some evidence for an over-representation of overlapping genes among specific functional categories, that is, imprinted genes and DNA repair genes [1]. Twenty-two out of 58 known imprinted murine genes are transcribed from both strands. Frequently, one partner transcribes a noncoding RNA. Antisense transcripts may regulate imprinting states of the sense promoter (*Kcnq1*/*Kcnq1ot1*) or may induce dsRNA-based gene silencing as proposed for *Ifd2R*/*Air*. About 20% of known human DNA repair genes overlap either convergently or divergently in an antiparallel arrangement [1].

Mammalian mRNAs that form sense-antisense pairs frequently exhibit reciprocal expression patterns, but permanent coexpression of sense and antisense transcripts can also occur in some tissues, although it is difficult to prove that both genes are transcribed in the same cell. Coexistence of sense and antisense transcripts may indicate a stabilizing effect of dsRNA, or it may depict cases where RNA duplex formation is impaired as a result of secondary structures, or because sense and antisense transcripts or the enzymes required for duplex formation are separated by compartmentalization [13].

To gain an insight into the existence and role of overlapping antisense pairs in plants, we have screened the *Arabidopsis thaliana *genome for COPs with sense and antisense genes that encode a protein, and have compared the expression profile of the associated genes.

## Results

### Overlapping gene pairs in the *Arabidopsis *genome

**Figure 1:**
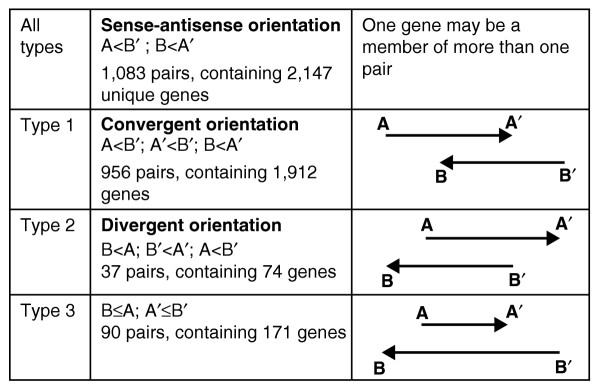
A comparison of the arrangements of overlapping gene pairs in *Arabidopsis thaliana*. A and A' label the start and end of the sense transcript, B' and B label the start and end of the antisense transcript. The total number of genes involved in group 1, 2 and 3 is 2,157, of which 2,147 are unique; the remaining 10 comprise four genes that are members of both group 1 and group 2 pairs, five genes that are members of both group 1 and group 3 pairs, and 1 gene that is a member of both a group 1 and group 3 pair.

**Figure 2:**
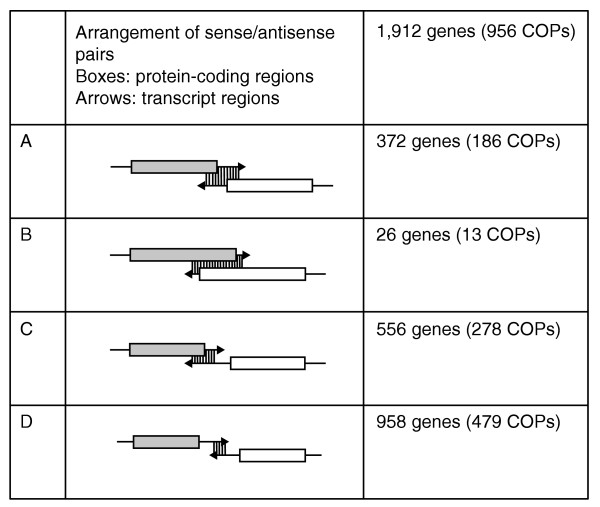
The organization of convergent overlapping gene pairs with respect to the protein coding capacity of the sense and antisense transcripts.

A screen of the *Arabidopsis *genome for protein-coding genes with overlapping orientations identified 1,083 groups containing a total of 2,147 overlapping genes. For 26 groups, the overlap involves three genes, and for 1,057 groups, two genes are arranged as overlapping pairs (Figure 1 and Additional data file 1). The majority of overlapping gene pairs are organized as COPs. The size of the overlapping region for these 956 COPs varies between 1 and 2,820 bp, with an average length of 431 bp. The genes are scattered around all five chromosomes with no obvious clustering bias (data not shown). Among the 1,912 COP genes, we found nine transposable elements. This is in contrast to the presence of 2,372 transposons among the 30,624 *Arabidopsis *genes. Transposons are therefore strongly under-represented among COPs, therefore transposon-derived antiparallel gene pairs are under heavy selective pressure. Among the 1,912 genes of the 956 COPs, 954 genes have antisense transcripts that extend into the open reading frame (ORF) region of the sense transcript (Figure a-c and Additional data file 2) but the ORF regions of the sense and antisense transcript overlap for only 13 COPs (Figure 2a and Table 1).

To examine the degree of sequence conservation for COPs sense and antisense genes, we used BLAST to search for homologs of each COPs gene. For a subset of 242 genes, we can define 89 homology groups with 2-11 members. The proteins encoded by the sense members of each group are at least 20% identical, with an E-value less than 0.001. An analysis of the degree of sequence conservation among family members showed very low conservation among the coding or promoter regions of the antisense partners of homologous sense genes. With the exception of the largest family, the promoter regions of homologous sense genes are also poorly conserved (Table 2 and Additional data files 4, 5 and 6). With a few exceptions, possibly representing relatively recent duplications, the data indicate that homologous sense genes do not in general have homologous antisense partners.

### COPs gene-expression profiles

To analyze the transcriptional activity of COPs sense and antisense genes, we used the GSE636 annotated gene-expression database [14,15], which provides expression data for 1,866 COPs genes in suspension culture, in 7-day old seedlings, in roots and in flowers. If antisense arrangements are predominantly responsible for dsRNA-mediated transcript degradation, we would expect that a significant proportion of COPs genes would be under-represented among the transcript pool. For the total pool of 26,939 genes represented in the GSE636 database, we find a representation of 49.8-53.1% of these genes among the detectable transcripts (Table 3). Of the 1,866 COPs genes represented among this pool, 63.4-67.9% are expressed, which argues against a specific depletion of COPs transcripts in any of the four sample tissues.

This assumption is further supported by the lack of any bias against the joint expression of sense and antisense copies from the same COP. We can calculate a theoretical value for the joint expression of a sense and an antisense member of the same COP based on the representation of the COP genes in the transcriptional pool. If, for example, the probability that a COP gene is expressed in flowers is 67.9%, the probability that any two COP genes are jointly expressed is 67.9% × 67.9%. The expected value of 46.1% matches the observed value of 45.6% determined for the joint expression of both genes of a COP (Table 3). We observe a similar match for the other tissues, which suggests that there is no bias against the joint expression of both COP partner genes. For about 20% of all COPs, both members are jointly expressed in all tissues tested.

We also examined the microarray data provided by the Nottingham *Arabidopsis *Stock Centre (NASC) [16]. Table 4 compiles 21 Affymetrix ATH1 arrays for seven different *Arabidopsis *tissues based on three replicates for each assay. The datasets were retrieved by searching for BioSource_ID on [17].

Although the expression probabilities of both the total gene pool and the COPs gene pool differ significantly among individual tissues, the expected and observed values for the coexpression of COPs sense and antisense genes again match, reinforcing the lack of any indication of a transcript degradation mechanism (Table 4). To assess whether transcript degradation depends on a specific experimental condition, we assembled the expression data for all 1,437 microarrays that were available. For each microarray, we calculated the proportion of transcripts that were expressed, both for the total gene pool comprising 22,746 genes and for the 1,596 COPs genes represented in the total pool. A depletion of COPs-specific transcripts for any of the 1,437 microarrays should result in a significant reduction of the expressed COPs pool. We do not find a single case where the COPs genes are under-represented among the transcripts detectable in an array experiment. Compared to the transcriptional activity of the whole gene pool, the transcriptional activity of the COPs gene pool is between 1.008 and 1.485 times higher. This argues against a specific reduction in COPs gene activity under any of the experimental conditions used for the array experiments (Additional data file 7).

### Indications for a role of antisense transcripts in sense transcript splicing

Among the 1,912 COPs genes we find a considerable bias for splicing. Of the COPs genes, 1,723 (90.1%) are spliced, while among a total of 30,624 *Arabidopsis *genes only 21,157 (69.1%) genes are spliced. If antisense transcripts played a role in sense transcript alternative splicing, we would expect an enrichment among COPs genes in alternative splicing. Out of the total pool of 30,624 genes, 7.6% encode more than one transcript, which are all alternatively spliced. This proportion rises to 14.5% among the 1,912 COPs genes (Table 5). This increase is even more pronounced in the rice genome, where it increases from 4.4% to 20.9% COPs.

To assess if the over-representation of multiple transcripts among COPs was linked to a variation in splicing, transcriptional start site (TSS) or polyadenylation, we analyzed the representation of these modifications among the spliced COPs genes. The results showed a positive bias for alternative splicing (Table 5). Interestingly, this bias was restricted to COPs with an antisense transcript that overlaps the intron region of the sense transcript (Table 6). Moreover, among the COPs with antisense transcripts overlapping at least 40 bp of the sense transcript, we also detected a positive bias for a variation of the polyadenylation sites (Table 6), whereas no positive or negative bias was observed for TSS variation (see Additional data file 8).

**Figure 3:**
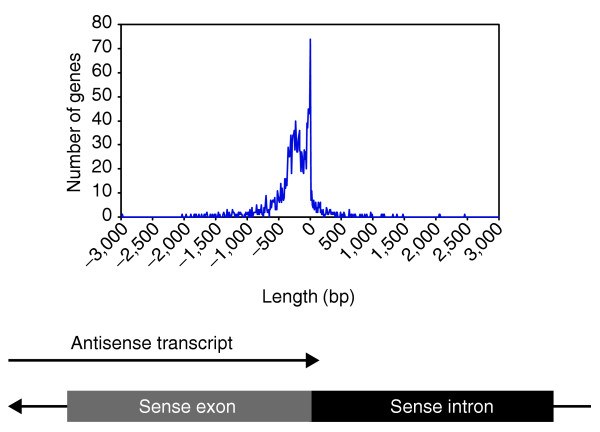
Illustration of the distance between the end of the antisense transcript and the last intron-exon boundary of the sense transcript. Negative values refer to a termination of the antisense transcript 5' to the intron-exon boundary.

For 146 genes, the antisense transcript terminates within 10 bp away from the intron-exon boundary of the sense transcript (Figure 3 and Additional data file 3). The proportion of alternatively spliced genes among this group increases to 21.2%. We tested whether these COPs genes were specifically prone to alternative splicing of the final exon but could not find any evidence for this assumption (see Additional data file 8).

Overall, the enhanced likelihood that members of overlapping gene pairs contain introns, the enrichment in genes encoding alternatively spliced transcripts, and the increased frequency of alternatively spliced and variably polyadenylated transcripts when an intron overlaps with an antisense transcript, suggest that, at least for the majority of overlapping gene pairs, the antisense transcript could play a role in the regulation of splicing and/or polyadenylation.

## Discussion

We have characterised the organization and expression profiles of 956 convergent overlapping gene pairs of *A. thaliana *to assess the potential molecular mechanisms associated with this unusual gene organization, which provided the opportunity for dsRNA formation as a result of the annealing of a sense and antisense transcript.

In animal genomes especially, a number of different mechanisms have been described that involve dsRNA formation. dsRNA formation can interfere with biological activities that require binding of RNA or proteins to the transcript [13]. This may include processes such as RNA splicing, editing, transport, degradation or translation. Alternatively, dsRNA could function as a trigger for an RNAi process, providing a target for Dicer enzymes, and be specifically degraded to small interfering RNA (siRNA) molecules [18]. The latter mechanism would lead to mutual destruction of the sense and antisense transcripts, whereas antisense transcript-mediated effects on sense RNA processing would not necessarily alter the primary transcript levels, although they could still influence the potential for primary transcript expression.

As in other species [1,19], the majority of overlapping gene pairs in *A. thaliana *are arranged in a convergent orientation. Only a very small group of 13 out of 956 COPs show an overlap between the ORFs of the sense and antisense transcripts, which probably reflects the associated evolutionary stress of such an arrangement, as any mutation in the overlapping region would affect both genes. For 50.1% of COPs the sense-antisense overlap does not include any protein-coding region, which makes it unlikely that in this subgroup of COPs the antisense transcript plays a role in regulating the coding region of the sense gene. Antisense transcripts for the members of this group are more likely to jointly use bidirectional poly(A) signals [6] or to regulate transcript stability [7].

We do observe a very high level of joint expression of sense and antisense transcript from overlapping gene pairs. This is in marked contrast to data from *Plasmodium falciparum*, where only 5% of sense-antisense loci show joint expression [20] and thus support models for a direct regulation of sense transcript by antisense expression via dsRNA degradation. In contrast, the relatively high expression frequency of COPs in *Arabidopsis*, and the joint presence of sense and antisense transcripts in the same tissue, do not support a dsRNA degradation model. Even a detailed analysis of 1,437 microarrays does not imply that under any conditions or for any specific tissue the COPs gene pool is significantly depleted. While dsRNA-based transcript degradation may occur for some COPs, our data suggest that for the majority of COPs, antisense expression is not linked to transcript degradation pathways.

An interesting observation, which may hint at an alternative interference mechanism between sense and antisense transcripts, is the significant bias for COPs to be spliced, and the enrichment among COPs of alternatively spliced transcripts. These features may indicate a role for antisense transcripts in alternative splicing. Such a mechanism would resemble the effect of antisense expression for the thyroid hormone receptor gene, *erbAα*, which leads to alternative RNA processing [5]. The bias for antisense transcripts to terminate close to the final intron-exon boundary remains a mystery. One could assume that the termination of the antisense transcript near the final sense intron-exon boundary might reflect a selection for antisense transcripts that interfere with splicing of the last intron. However, we could not observe any positive bias for such events among this COPs gene group.

The assumption that antisense transcripts can interfere with splicing events is further supported by the observation that overlaps between antisense transcripts and a sense intron region generate a bias for alternative splicing and also for polyadenylation variation. This may reflect a linkage between these two mechanisms, which has been demonstrated for animal systems where polyadenylation and the splicing of the final intron especially can be coupled [21].

During the review process of this paper, a similar study by Wang and co-workers [22] was published. The main differences between the two studies are in the sets of overlapping genes considered, and the nature of the experimental evidence of gene expression. While we consider only gene pairs where both partners show evidence of protein-coding capacity, Wang and co-workers also considered cases where at least one transcript may not be protein coding. We base our studies of expression on two large microarray datasets, while Wang and co-workers use data from a massively parallel signature sequencing (MPSS) study. While we find no evidence in the microarray data of exclusive transcription relationships for COPs gene pairs, the MPSS evidence of exclusive transcription of the gene pairs in the Wang study is clear. This apparent contradiction may be explained by differences in the gene sets studied, particularly as expression data is only available for a subset of the genes in each study, or by differences in the quality of the expression data. Nevertheless, the two studies taken together give evidence of various significant biological consequences of gene overlaps, including effects on sense gene splicing or polyadenylation (our study) and coexpression of gene pairs [22].

It is important to remember that in our study, we have concentrated on a specific subgroup of convergently overlapping genes, with both sense and antisense transcripts encoding an ORF. Among this group there may be an over-representation of gene pairs for which sense and antisense transcription jointly regulate the production efficiency of both proteins, for example via the common use of a bidirectional polyadenylation region, or by co-editing of both strands associated as dsRNA. Such mechanisms would require the joint transcription of both genes in the same tissue, and our data do suggest that sense and antisense transcripts are frequently coexpressed. On the other hand, one would assume that co-regulation would preferentially be used for genes encoding proteins that are linked in their biological role. One would therefore expect a high degree of conservation for both proteins among homologous COPs, whereas our data show that COPs with homologous proteins encoded by their sense genes do not show the same conservation for the proteins encoded by their antisense genes.

The selection of sense/antisense transcripts with coding capacity may also be the reason for the lack of an indication of dsRNA-based degradation of COPs. A considerable proportion of overlapping antisense genes are noncoding RNAs [23] or are *trans*-NATs transcribed from different genetic loci [3]. These overlapping transcript types may contain a much higher proportion of genes regulated by transcript degradation than the COPs analyzed in this study. A final confirmation of the role of dsRNA for individual genes will require a more detailed experimental analysis. Our analysis should, however, provide a useful first step in defining distinct groups of COP genes as a basis for a more detailed molecular characterization.

## Conclusion

The *Arabidopsis *genome contains 956 COPs with coding capacity that have the potential to form dsRNA. In contrast to data from other species, a comparative expression analysis indicates that sense and antisense transcripts of COPs loci can coexist in the same tissue at the same frequency as the transcripts of any other unlinked genes, with no indication of specific degradation of such sense-antisense transcript pairs. This observation does not exclude the presence of dsRNA degradation pathways for individual loci, but it refocuses the attention on alternative roles for natural antisense transcripts in plants, preferentially those that do not lead to an overall change in transcript levels but rather affect transcript processing or localization. In line with this view, we observe a high proportion of intron-containing genes in COPs, in both *Arabidopsis *and rice, and an enrichment for genes with alternatively spliced transcripts, indicative of a role for some COP antisense transcripts in splicing modification. In addition, we detect a potential link between alternative splicing and poly(A) site variation. This work provides a set of databases for COPs, based on the degree of sense-antisense overlap and expression, which should provide a basis for the selection of individual candidate loci for a detailed molecular analysis of the different dsRNA pathways.

## Materials and methods

### Analysis of overlapping transcripts

All *Arabidopsis *genome information, including gene ID (AGI code), transcript orientation, and gene and exon position coordinates of transcripts with coding regions, was downloaded from The *Arabidopsis *Information Resource (TAIR) ftp website [24]. These data were stored in a MySQL database [25] designed for general genomic analysis, and the overlapping transcript analysis was implemented with custom SQL language queries and Perl scripts using the Perl DBI module. The rice genome data were obtained from [26,27].

### Analysis of gene variation

Genes with more than one transcript were further analyzed for variation in TSS, polyadenylation site, and alternative splicing. TSS variation was detected by comparing the starting genomic positions of the first exons of genes with more than one transcript, and variation in the polyadenylation site by comparing the ends of the last exons. Genes with more than one transcript that had identical TSSs and polyadenylation sites, or had different numbers of exons, were considered as alternatively spliced. Genes with more than one transcript that had the same number of exons and variation in TSS and polyadenylation site, underwent comparison of their intron boundaries to detect alternative splicing. To detect alternative splicing in the last intron, alternatively spliced transcripts underwent comparison of the borders of their last intron.

### Hypergeometric distribution

*p*-values for over- or under-representations of genes were calculated as the upper or lower tail of the hypergeometric distribution *p*(*x *≥ X) or *p*(*x *≤ *X*), respectively, where *p*(*x;N,R,k*) = *C*(*R,x*)*C*(*N *- *R,k *- *x*)/*C*(*N,k*). Here *p *refers to the probability that a list of *k *genes should contain *x *genes with a particular property (for example, alternative splicing), when the list has been selected randomly without replacement from a set of *N *genes in which *R *genes exhibit the same property. *C*(*n,m*) is the number of distinct combinations of m objects that can be drawn from a set of *n *objects. The hypergeometric distribution was calculated with the *R *package [28].

### Microarray data

*Arabidopsis *microarray data were obtained from two sources. The dataset from Gene Expression Omnibus [14,15] with accession number GSE636 is a collection of microarray experiments using high-density oligonucleotide arrays. It contains transcriptional activity information (detection call only) for the complete set of all protein-coding genes in different tissues. The Affymetrix ATH1 array data were acquired using the Nottingham *Arabidopsis *Stock Centre (NASC) AffyWatch service [16]. In NASC's datasets, including 1,437 arrays for 93 experimental purposes, the transcription information for each gene consists of detection call and signal value, as calculated from the Affymetrix MAS 5.0 analysis algorithms [29]. The analysis of expression data reported in Results was achieved using a combination of Perl script processing and Microsoft Excel spreadsheet analysis.

### Coding region and upstream sequence similarity analysis of homologous COP genes

The COPs genes were clustered on the basis of their protein sequences with a 20% similarity threshold using the program BLASTclust [30]. The similarity of the associated coding and upstream regulatory regions within each cluster were tested by pairwise searches using BLAST2P [31].

## Additional data files

The following additional files are available with the online version of this paper. Additional data file 1 is a supplement to Figure 1 listing all overlapping genes with ID, annotation and size of overlapping region. Additional data file 2 is a supplement to Figure 2 classifying 1,912 COPs genes according to their overlapping regions. Additional data file 3 is a supplement to Figure 3, calculating the antisense transcript end position in relation to the sense intron-exon boundary for the 956 COPs pairs. Additional data file 4 is a supplement A to Table 2, listing 242 COPs member genes of 89 COPs families. Additional data file 5 is a supplement B to Table 2, comparing homology among 1 kb promoter regions of COPs family members. Additional data file 6 is a supplement C to Table 2, comparing homology among sense and antisense encoded proteins for members of 89 COPs families. Additional data file 7 is a supplement to Table 4, showing expression analysis of the total gene pool and the COPs gene pool for 1,437 microarray experiments. Additional data file 8 is a supplement to Table 6, including a correlation analysis of alternative splicing, TSS variation and polyadenylation variation for COPs with respect to the termination of the antisense transcript in relation to the sense intron-exon boundary.

## Supplementary Material

Additional File 1 Supplement to Figure 1. List of all overlapping genes with ID, annotation and size of overlapping region. List of all overlapping genes with ID, annotation and size of overlapping region Click here for file

Additional File 2 Supplement to Figure 2. Classification of 1,912 COPs genes according to their overlapping regions. Classification of 1,912 COPs genes according to their overlapping regions Click here for file

Additional File 3 Supplement to Figure 3. Calculation of the antisense transcript end position in relation to the sense intron-exon boundary for the 956 COPs pairs. Calculation of the antisense transcript end position in relation to the sense intron-exon boundary for the 956 COPs pairs Click here for file

Additional File 4 Supplement A to Table 2. 242 COPs member genes of 89 COPs families. 242 COPs member genes of 89 COPs families Click here for file

Additional File 5 Supplement B to Table 2. Comparison of homology among 1 kb promoter regions of COPs family members. Comparison of homology among 1 kb promoter regions of COPs family members Click here for file

Additional File 6 Supplement C to Table 2. Homology comparison among sense and antisense encoded proteins for members of 89 COPs families. Homology comparison among sense and antisense encoded proteins for members of 89 COPs families Click here for file

Additional File 7 Supplement to Table 4. Expression analysis of the total gene pool and the COPs gene pool for 1,437 microarray experiments. Expression analysis of the total gene pool and the COPs gene pool for 1,437 microarray experiments Click here for file

Additional File 8 Supplement to Table 6. Correlation analysis of alternative splicing, TSS variation and polyadenylation variation for COPs with respect to the termination of the antisense transcript in relation to the sense intron-exon boundary. Correlation analysis of alternative splicing, TSS variation and polyadenylation variation for COPs with respect to the termination of the antisense transcript in relation to the sense intron-exon boundary Click here for file

## Figures and Tables

**Table 1:** COPs with sense-antisense overlaps within the coding regions

Sense gene ID	Annotation	Antisense gene ID	Annotation	ORF overlap (bp)
AT1G08260	DNA-directed DNA polymerase epsilon catalytic subunit, putative	AT1G08270	Expressed protein	45
AT1G52010	Mutator-like transposase family	AT1G52020	Pseudogene, Ulp1 protease family	44
AT1G52087	Hypothetical protein	AT1G52090	Hypothetical protein	72
AT1G68935	Expressed protein	AT1G68940	Armadillo/beta-catenin repeat protein-related/U-box domain-containing protein	698
AT2G12855	Gypsy-like retrotransposon family	AT2G12860	Gypsy-like retrotransposon family	116
AT2G19330	Leucine-rich repeat family protein	AT2G19340	Membrane protein, putative	141
AT3G59940	Kelch repeat-containing F-box family protein	AT3G59950	Autophagy 4b (APG4b)	10
AT4G02200	Drought-responsive family protein	AT4G02210	Expressed protein	13
AT4G21366	S-locus protein kinase-related	AT4G21370	S-locus protein kinase, putative	72
AT4G29830	Transducin family protein/WD-40 repeat family protein	AT4G29840	Threonine synthase, chloroplast	587
AT5G18210	Short-chain dehydrogenase/reductase (SDR) family protein	AT5G18220	Glycosyl hydrolase family 17 protein	6
AT5G28232	Mutator-like transposase family	AT5G28235	Ulp1 protease family protein	29
AT5G48200	Hypothetical protein	AT5G48205	Hypothetical protein	334

**Table 2:** Homology assessment for 89 COPs families that contain 2-11 family members

Number of COPs family	Number of family members	Sense-gene-encoded proteins with a similarity E-value < 0.001	1 kb sense promoter regions with a similarity E-value < 0.001	Antisense-gene-encoded proteins with a similarity E-value < 0.001	1 kb antisense promoter regions with a similarity E-value < 0.001
1	11	11	7	2	2
2	8	8	2	0	0
3	8	8	0	0	2
4	7	7	0	0	0
5-6	7	7	2	0	0
7	6	6	2	2	0
8-9	5	5	0	0	0
10	4	4	2	0	0
11-12	4	4	0	0	0
13-14	4	4	0	2	0
15	4	4	2	2	0
16-21	3	3	0	0	0
22-72	2	2	0	0	0
73-75	2	2	2	0	0
76-77	2	2	0	2	2
78-89	2	2	0	2	0

The numbers refer to the family members that share sequence similarity of an E-value below 0.001 with at least one other family member. Among the COPs families, the homology is well conserved among sense-gene-encoded proteins, while sequence conservation is rare among antisense-gene-encoded proteins. With the exception of family 1 sense gene promoters, the homology is also poorly conserved among promoter regions of sense and antisense genes.

**Table 3:** Expression analysis of 1,866 COPs genes based on expression data from the GSE636 annotated gene-expression database

Tissue	% of expressed genes among 26,939 *Arabidopsis *genes	% of expressed genes among 1,866 overlapping COPs genes	% of COPs with jointly expressed sense and antisense genes (observed value)	% of COPs with jointly expressed sense and antisense genes (expected value)
Flowers	52.5%	67.9%	45.6%	46.1%
Roots	51.4%	63.4%	38.5%	40.2%
Suspension culture	53.1%	66.3%	42.7%	44.0%
7 day old seedlings	49.8%	64.1%	40.4%	41.1%

**Table 4:** Expression of 1,596 COPs genes based on the NASC microarray database

Tissue	% of expressed genes among 22,746 *Arabidopsis *genes	% of expressed genes among 1,596 overlapping COPs genes	% of COPs with jointly expressed sense and antisense genes (observed value)	% of COPs with jointly expressed sense and antisense genes (expected value)
Flowers	62.9%	82.5%	67.7%	68.1%
Pollen	31.7%	36.0%	12.3%	13.0%
Seedlings, green parts	57.3%	76.7%	57.8%	58.8%
Cotyledons	55.7%	75.3%	56.1%	56.7%
Leaves	55.6%	74.8%	54.5%	56.0%
Roots	62.6%	76.9%	58.3%	59.1%
Hypocotyl	62.9%	82.1%	66.8%	67.4%

**Table 5:** Representation of spliced genes among COPs, and correlation analysis for transcript modifications among these genes

	Total genes	COPs	*p*-value
COP genes show a strong positive bias for splicing			
Total	30,624	1,912	
Spliced	21,157 (69.1%)	1,723 (90.1%)	4.7e-113
			
Spliced COP genes show a positive bias for alternative splicing			
Spliced	21,157	1,723	
Alternatively spliced	2,331 (11.0%)	268 (15.6%)	1.3e-9
			
Alternatively spliced COP genes do not show a significant bias for alternative splicing at the last intron, TSS variation or polyadenylation site variation			
Alternatively spliced	2,331	268	
Last intron alternative splicing	1,662 (71.3%)	195 (72.8%)	0.31
TSS variation	1,424 (61%)	158 (59.0%)	0.24
Polyadenylation site variation	1,019 (43.7%)	107 (39.9%)	0.10

**Table 6:** Analysis of preferences for alternative splicing and polyadenylation site variation among spliced COPs genes, in dependence of the termination site of the antisense transcript

Spliced COP genes with an antisense transcript not overlapping a sense transcript intron region, show a significant negative bias for alternative splicing			
	COPs genes	COPs with antisense gene ending 3,000-0 bp before the sense I/E boundary	*p*-value
Spliced genes	1,723	1,497	
Alternatively spliced genes	268 (15.6%)	217 (14.5%)	0.0018
			
Spliced COPs genes with an antisense transcript overlapping a sense transcript intron region, show a significant positive bias for alternative splicing			
	COPs genes	COPs with an antisense gene ending 0-3,000 bp behind the sense I/E boundary	*p*-value
Gene with splicing	1,723	226	
Alternative splicing	268 (15.6%)	51 (22.6%)	0.0018
			
	COPs genes	COPs with an antisense gene ending > 40 bp behind the sense I/E boundary	*p*-value
Gene with splicing	1,723	129	
Alternative splicing	268 (15.6%)	35 (27.1%)	0.00032
			
Alternatively spliced COPs sense genes with an antisense transcript ending more than 40 bp behind their last I/E boundary, show a positive bias for polyadenylation site variation			
	COPs genes	COPs with an antisense gene ending > 40 bp behind the sense I/E boundary	*p*-value
Alternatively spliced	268	35	
Polyadenylation site variation	107 (39.9%)	25 (71.4%)	5.5e-05
