# Supplementary material for: Natural antisense transcripts with coding capacity in Arabidopsis may have a regulatory role that is not linked to double-stranded RNA degradation
Source: Genome Biol. 2005 Jun 1;6(6):R51. doi: 10.1186/gb-2005-6-6-r51 (PMC1175971; doi:10.1186/gb-2005-6-6-r51)
Supplement: Additional File 8 — Supplement to Table 6. Correlation analysis of alternative splicing, TSS variation and polyadenylation variation for COPs with respect to the termination of the antisense transcript in relation to the sense intron-exon boundary. Correlation analysis of alternative splicing, TSS variation and polyadenylation variation for COPs with respect to the termination of the antisense transcript in relation to the sense intron-exon boundary [file gb-2005-6-6-r51-S8.pdf]

Supplement to table 6: Correlation analysis of alternative splicing, TSS variation and polyadenylation variation for COPs with respect to the termination of the antisense transcript in relation to the sense intron/exon boundary.

I. COPs with no overlap between the antisense transcript and the sense intron region

Spliced COPs genes with an antisense transcript not overlapping a sense transcript intron region, show a significant negative bias for alternative splicing.			
	COPs genes	COPs with antisense gene ending 3000 - 0 bp before the sense I/E boundary	p-value
Gene with splicing	1723	1497	
Alternative splicing	268 (15.6%)	217 (14.5%)	0.0018

Alternatively spliced COPs genes with an antisense transcript not overlapping a sense transcript intron region, show no significant bias for alternative splicing at the last intron, for TSS variation or polyadenylation site variation.			
	COPs genes	COPs with antisense gene ending 3000 - 0 bp before the sense I/E boundary	p-value
Alternatively spliced	268	217	
Last intron alternatively spliced	195 (72.8%)	154 (71.0%)	0.12
TSS variation	158 (59.0%)	133 (61.3%)	0.075
Polyadenylation site variation	107 (39.9%)	80 (36.9%)	0.026

II. COPs with an overlap between the antisense transcript and the sense intron region

Spliced COPs genes with an antisense transcript overlapping a sense transcript intron region, show a significant positive bias for alternative splicing.			
	COPs genes	COPs with an antisense gene ending 0-3000 bp behind the sense I/E boundary	p-value
Gene with splicing	1723	226	
Alternative splicing	268 (15.6%)	51 (22.6%)	0.0018

Alternatively spliced COPs sense genes with an antisense transcript overlapping a sense transcript intron region, show no significant bias for alternative splicing at the last intron, for TSS variation or polyadenylation site variation.			
	COPs genes	COPs with an antisense gene ending behind the sense I/E boundary	p-value
Alternatively spliced	268	51	
Last intron alternatively spliced	195 (72.8%)	41 (80.4%)	0.12
TSS variation	158 (59.0%)	25 (49.0%)	0.075
Polyadenylation site variation	107 (39.9%)	27 (52.9%)	0.026

Spliced COPs sense genes with an antisense transcript ending more than 40bp behind their last I/E boundary, show a significant positive bias for alternative splicing.			
	COPs genes	COPs with an antisense gene ending >40bp behind the sense I/E boundary	p-value
Gene with splicing	1723	129	
Alternative splicing	268 (15.6.0%)	35 (27.1%)	0.00032

Alternatively spliced COPs sense genes with an antisense transcript ending more than 40bp behind their last I/E boundary, show no significant bias for alternative splicing at the last intron or TSS variation but have positive bias for polyadenylation site variation.			
	COPs genes	COPs with an antisense gene ending >40bp behind the sense I/E boundary	p-value
Alternatively spliced	268	35	
Last intron alternatively splicing	195 (72.8%)	31 (88.6%)	0.016
TSS variation	158 (59.0%)	15 (42.8%)	0.030
Polyadenylation site variation	107 (39.9%)	25 (71.4%)	5.5e-05

III. COPs with an antisense transcript ending in the close vicinity of the sense intron/exon boundary

Spliced COPs sense genes with an antisense transcript ending near their last I/E boundary, show no significant bias for alternative splicing.			
	COPs genes	COPs with an antisense gene ending within the -10 to 10bp region of the last I/E boundary	p-value
Spliced	1723	148	
Alternatively spliced	268 (15.6%)	31 (20.9%)	0.042

Alternatively spliced COPs sense genes with an antisense transcript ending near their last I/E boundary, show no significant bias for alternative splicing at the last intron or for TSS variation but show a negative bias for polyadenylation site variation.			
	COPs genes	COPs<br>Antisense gene end at -10~10 of the last I/E	p-value
Alternatively spliced	268	31	
Last intron alternative splicing	195 (72.8%)	17 (54.8%)	0.018
TSS variation	158 (59.0%)	17 (54.8%)	0.38
Polyadenylation site variation	107 (39.9%)	5 (16.1%)	0.0026
